# Genetic Variations in the Vitamin D Receptor Predict Type 2 Diabetes and Myocardial Infarction in a Community-Based Population: The Tromsø Study

**DOI:** 10.1371/journal.pone.0145359

**Published:** 2015-12-23

**Authors:** Ieva Zostautiene, Rolf Jorde, Henrik Schirmer, Ellisiv Bøgeberg Mathiesen, Inger Njølstad, Maja-Lisa Løchen, Tom Wilsgaard, Ragnar Martin Joakimsen, Elena Kamycheva

**Affiliations:** 1 Tromsø Endocrine Research Group, Department of Clinical Medicine, UiT The Arctic University of Norway, Tromsø, Norway; 2 Division of Internal Medicine, University Hospital of North Norway, Tromsø, Norway; 3 Department of Clinical Medicine, UiT The Arctic University of Norway, Tromsø, Norway; 4 Division of Cardiothoracic and Respiratory Medicine, University Hospital of North Norway, Tromsø, Norway; 5 Brain and Circulation Research Group, Department of Clinical Medicine, UiT The Arctic University of Norway, Tromsø, Norway; 6 Department of Neurology and Neurophysiology, University Hospital of North Norway, Tromsø, Norway; 7 Department of Community Medicine, UiT The Arctic University of Norway, Tromsø, Norway; National Cancer Center, JAPAN

## Abstract

**Background:**

Though the associations between low serum 25-hydroxyvitamin D (25(OH)D) levels and health outcomes such as type 2 diabetes (T2D), myocardial infarction (MI), cancer, and mortality are well-studied, the effect of supplementation with vitamin D is uncertain. This may be related to genetic differences. Thus, rs7968585, a single nucleotide polymorphism (SNP) of the vitamin D receptor (*VDR*), has recently been reported as a predictor of composite health outcome. We therefore aimed to evaluate whether rs7968585 predicts separate clinical outcomes such as T2D, MI, cancer, and mortality in a community-based Norwegian population.

**Methods and Findings:**

Measurements and DNA were obtained from the participants in the Tromsø Study in 1994–1995, registered with the outcomes of interest and a randomly selected control group. The impact of the rs7968585 genotypes was evaluated with Cox proportional hazards. A total of 8,461 subjects were included among whom 1,054 subjects were registered with T2D, 2,287 with MI, 3,166 with cancer, and 4,336 with death. Mean follow-up time from birth was 60.8 years for T2D and MI, 61.2 years for cancer, while mean follow-up time from examination date was 16.5 years for survival. Mean serum 25(OH)D levels did not differ across the rs7968585 genotypes. With the major homozygote genotype as reference, the minor homozygote subjects had hazard ratios of 1.25 (95% CI 1.05–1.49) for T2D and 1.14 (1.02–1.28) for MI (*P* = 0.011 and 0.023, respectively, without the Bonferroni correction). No significant interaction between serum 25(OH)D status and the rs7968585 genotype was found for any of the endpoints.

**Conclusions:**

The *VDR*-related SNP rs7968585 minor allele is a significant and positive predictor for T2D and possibly for MI. Since the functional mechanism of this SNP is not yet understood, and the association with T2D is reported for the first time, confirmatory studies are needed.

## Introduction

Vitamin D is a biologically active substance important not only for maintenance of calcium homeostasis but also for several nonskeletal metabolic pathways [1]. Despite the association between low serum 25-hydroxyvitamin D (25(OH)D) levels and increased risk of type 2 diabetes (T2D), cardiovascular diseases, cancer, and mortality [1–5], intervention studies with vitamin D thus far have been inconclusive [6]. Possibly, this may be due to genetic differences in vitamin D metabolism [7].

The vitamin D receptor (VDR) has been identified in most of the body’s tissues, including pancreatic islets, myocardium, and fibroblasts [8, 9]. The activated VDR regulates approximately 3% of all genes [10, 11] and may protect against diabetes by regulating insulin secretion and resistance and by reducing inflammatory damage to the pancreatic islets [12, 13]. Furthermore, the VDR regulates calcium homeostasis, may act antiatherosclerotic, may suppress the renin system, and may protect against myocardial hypertrophy and heart failure as well as coronary artery disease (CAD) [1, 14, 15]. In cancer pathogenesis, the activated VDR is suggested to act antiproliferatively and to regulate cell differentiation and apoptosis [16, 17]. Thus, the role of the VDR and its individual variations, instead of the serum concentration of vitamin D metabolites *per se* might be important in the pathogenesis of diabetes, myocardial infarction (MI), cancer, and mortality.

Many recent studies have focused on *VDR* polymorphisms as risk factors for these health outcomes, and some *VDR* single nucleotide polymorphisms (SNPs) are reported to be associated with risk of T2D [18], several forms of cancer including breast, prostate cancer, and malignant melanoma [19, 20], cancer-related mortality [21] and CAD [22–25], but not overall mortality [26].

Recently, the association between the common *VDR* gene variant rs7968585 and risk of a composite health outcome including hip fracture, MI, cancer, and mortality has been reported in subjects with low serum 25(OH)D levels [27]. However, in a subsequent study no associations between rs739837, a SNP in high linkage disequilibrium (LD) with rs7968585, and cardiometabolic outcomes were found [28].

The Tromsø Study is a large community-based study in northern Norway with repeated surveys every 6–7 years [29]. In the fourth survey performed in 1994–1995, lifestyle information and blood samples were collected from more than 27,000 subjects [30], and detailed endpoint registers were created. We were therefore able to evaluate the reported association between the rs7968585 genotypes, serum 25(OH)D and separate clinical outcomes such as T2D, MI, cancer, and mortality. Furthermore, since we also had genotype data on five other VDR SNPs (FokI (rs2228570/rs10735810), BsmI (rs1544410), TaqI (rs731236), ApaI (rs7975232) and Cdx2 (rs11568820)), we used the same approach to evaluate their association with the above mentioned outcomes.

## Materials and Methods

### The Tromsø Study

The Tromsø study initiated in 1974 is a longitudinal, community-based multipurpose Norwegian study focusing on lifestyle-related diseases [29]. It is performed in the Tromsø municipality, which is situated at 69° North with a current population of about 72,000 subjects. In the fourth survey in 1994–1995, all individuals aged 25 years or older were invited to participate; 77% participated, comprising a total of 27,158 subjects [30] of whom 26,956 were included in our analysis. A group of subjects were invited for a second visit for more extensive clinical examination and measurements, and 7,965 subjects, or 78% of the invited, participated [31].

### Selection of the study subjects

The selection of subjects for the genetic studies has been described in detail previously [32]. Briefly, the subjects were primarily selected for evaluation of genetic polymorphisms and clinical endpoints registered in the Tromsø Study, including T2D, MI, cancer, and death. Since these endpoints were of potential interest regarding genetic polymorphisms, and limited funding did not allow genetic analyses of the entire Tromsø Study cohort, a case—cohort design was used with randomly selected controls from the entire cohort who participated in the fourth survey [33]. Accordingly, genotyping was performed in all subjects included in the endpoint registers as well as the randomly selected controls and was successful for 11,752 subjects. Since this control group was randomly selected from the entire cohort, the group included numerous subjects with one or more endpoints. When analyzing a specific endpoint, subjects in the control group with that specific endpoint were moved to the case group. Therefore, the size of the control groups varied depending on the endpoint in question.

### Definition of T2D, MI, cancer, and death endpoints

The identification of subjects with endpoints has also previously been described in detail [34, 35]. In short, subjects with T2D were identified with self-reported diabetes questionnaires, elevated HbA_1c_ (≥6.5%), and a search in medical records and national registries. Differentiation between T1D and T2D was based on clinical judgment and blood tests (C-peptide, anti-glutamic acid decarboxylase (anti-GAD)) when available. Cases of unknown types of diabetes were excluded from the analyses.

Subjects with MI were identified by searching the related diagnoses in all available registries and records using the World Health Organization (WHO) Multinational MONItoring of Trends and Determinants in CArdiovascular Disease/MOnica Risk, Genetics, Archiving and Monograph (MONICA/MORGAM) diagnostic criteria [36]. Silent MIs defined with electrocardiogram only were not included as cases because of difficulties in determining the exact date of the event. The T2D and MI endpoints were included until the end of 2011 but completely updated until the end of 2010.

Cancer events were obtained from the Cancer Registry of Norway, with endpoints registered until the end of 2010. The whole cancer group was also divided into four subgroups according to location: breast, lung, colorectum, and prostate.

Information about death was retrieved from the Causes of Death Registry, and information about participants who moved out of the Tromsø area was obtained from the Norwegian Registry of Vital Statistics, updated until January 2013.

### Measurements

Baseline information was collected by physical examination, nonfasting blood samples, and self-administered questionnaires in 1994–1995. Blood pressure, weight, height, total cholesterol, and HbA_1c_ were measured/analyzed as previously described [37]. Information on current smoking and current physical activity was collected from these questionnaires. Physical activity was defined as the presence or absence of light or hard physical activity during leisure time. Body mass index (BMI) was calculated as weight divided by height squared.

Sera, collected during the second visit of the Tromsø Study in 1994–1995, were stored at –70°C, thawed in 2008, and analyzed for serum 25(OH)D using an automated clinical chemistry analyzer (Modular E170, Roche).

DNA was prepared with the manual isolation method from whole blood samples collected during the first visit. Genotyping was performed using the KASP (KBioScience Allele-Specific Polymorphism) SNP genotyping system as previously described [32].

### Statistical analyses

Distribution of the variables serum 25(OH)D, systolic blood pressure, BMI, total cholesterol, and HbA_1c_ were evaluated with skewness, kurtosis, and visual inspection of histogram and found normal except for HbA_1c_, which, however, could not be normalized with log transformation. Trends across the VDR SNP genotypes were evaluated with linear regression for continuous variables, using age, gender, and for 25(OH)D also season (months, using dummy variables), as covariates. For categorical variables, trends across genotypes were evaluated with the chi-square test with linear-by-linear association, and for HbA_1c_, the Kruskal—Wallis test was used for testing differences between the genotypes. Since serum 25(OH)D levels in smokers are overestimated when analyzed with the Modular E170 (Roche) [38], smokers and nonsmokers were analyzed separately unless otherwise specified. The Student’s t-test, Mann-Whitney U test, or Pearson’s chi-square test was used to compare variables in endpoint groups and their controls.

The genotype frequencies were evaluated for Hardy-Weinberg equilibrium using the chi-square test [39].

The hazard ratio (HR) of the VDR SNP genotypes for T2D, MI, cancer, and mortality was estimated with Cox regression with the major homozygote genotype used as the reference. Observation time was from birth until the event of interest for all endpoints except for death, where the observation time was from 1994.

In Model 1, for all endpoints, an adjustment for age and gender was made. In Model 2 for T2D and MI additional adjustment for data obtained in 1994/1995 on BMI, systolic blood pressure, total cholesterol, current smoking, and physical activity was performed. For MI, T2D was additionally included in Model 3. Since the effects of genotypes on cancer and mortality risk were not significant in any model regardless of covariates included, only Model 1 results were presented.

Additionally, serum 25(OH)D levels below or above the 20^th^ percentile as well as serum 25(OH)D used as a continuous variable were evaluated for association with T2D, MI, cancer, and death with age and gender as covariates. Similarly, interaction between serum 25(OH)D below or above the 20th percentile as well as 25(OH)D as a continuous variable and the VDR SNP genotypes were also tested with Cox regression with age and gender as covariates. In the analysis of a specific endpoint other endpoints were not included as covariates unless related to rs7968585 genotypes which was the case only for MI and T2D.

In the 20th percentile analyses, smokers and nonsmokers were analyzed together using month-specific 20th percentile cutoffs calculated separately for smokers and nonsmokers. When 25(OH)D was used as a continuous variable, smokers and nonsmokers were analyzed separately. These analyses with 25(OH)D were performed on incident cases only (observation time from Tromsø 4, 1994–1995).

The data are shown as mean ± standard deviation (SD). All tests are presented two-sided, and *P*-value <0.05 was considered statistically significant. The data were analyzed with IBM SPSS Statistics 22 (SPSS Inc., Chicago, IL, USA). The data are presented without correction for multiple analyses unless specified in the text if the Bonferroni correction was applied (*P*-value multiplied by a factor of 4 (four main endpoints)).

### Ethics

The study was approved by the Regional Committee for Medical and Health Research Ethics (REK Nord) (reference 2010/2913-4). Only participants with valid written consent were included.

## Results

The baseline characteristics of the entire Tromsø Study and the endpoint groups relevant for those successfully genotyped for rs7968585 are shown in Table 1. Serum 25(OH)D values both for smokers and nonsmokers were lower in the T2D and mortality groups than in the respective control groups. There were significantly more men and smokers in all case groups compared to the respective control groups. The rs7968585 genotype frequencies were not found to deviate.

**Table 1:** Baseline characteristics in 1994–1995 in the entire Tromsø Study population and in the endpoint groups related to the rs7968585 analyses. The Tromsø Study.

	Entire Tromsø Study population	T2D cases	T2D controls	MI cases	MI controls	Cancer cases	Cancer controls	Death cases	Death controls
N	26,956	1,054	3,509	2,287	3,166	3,166	3,137	4,336	2,109
Age (years) in 1994/95	46.9 ± 15.1	59.9 ± 12.2**	65.0 ± 12.7	63.0 ± 12.7*	63.9 ± 12.8	58.9 ± 13.5**	64.6 ± 13.0	66.9 ± 12.6**	58.1 ± 10.7
Sex (% females)	52.5	45.8**	57.5	35.7**	59.9	51.8**	59.0	48.9**	58.9
Current smokers (%)	32.7	31.4*	28.3	37.6**	27.6	36.8**	26.7	35.1**	28.0
Systolic BP (mmHg)	135 ± 21	152 ± 23**	148 ± 25	151 ± 24**	147 ± 24	143 ± 23**	149 ± 25	152 ± 25**	142 ± 22
BMI (kg/m^2^)	25.2 ± 3.9	28.8 ± 4.6**	25.6 ± 3.9	26.6 ± 4.0**	25.8 ± 4.1	25.7 ± 4.1*	25.9 ± 4.1	26.0 ± 4.3	26.0 ± 3.9
Total cholesterol (mmol/L)	6.05 ± 1.31	6.71 ± 1.22	6.68 ± 1.33	6.91 ± 1.27**	6.62 ± 1.32	6.45 ± 1.30**	6.71 ± 1.33	6.72 ± 1.32*	6.62 ± 1.30
HbA_1c_ (%)^a^	5.45 ± 0.66	6.27 ± 1.40**	5.39 ± 0.36	5.61 ± 0.83**	5.46 ± 0.67	5.50 ± 0.65	5.51 ± 0.79	5.61 ± 0.87**	5.43 ± 0.49
Serum 25(OH)D (nmol/L) smokers^b^	72.4 ± 20.0	66.6 ± 17.4**	73.9 ± 19.9	70.2 ± 18.8*	73.9 ± 20.4	72.6 ± 18.2	73.5 ± 20.2	70.4 ± 18.6**	75.3 ± 20.9
Serum 25(OH)D (nmol/L) nonsmokers^c^	52.4 ± 16.6	48.5 ± 16.3**	51.9 ± 16.3	51.6 ± 17.4	51.3 ± 16.1	52.7 ± 17.5*	50.9 ± 15.7	50.4 ± 17.2*	52.0 ± 15.8
Physical activity (% active)	54.6	32.2	30.7	32.1	32.5	36.5**	30.8	25.0**	39.8

^a^Measured only in those attending the second visit of the Tromsø Study in 1994–1995, N = 7,182.

^b^Measured only in those attending the second visit of the Tromsø Study in 1994–1995, N = 2,334

^c^Measured only in those attending the second visit of the Tromsø Study in 1994–1995, N = 4,826

*P<0.05,

**P<0.01 vs. respective control group; Student’s t-test, Mann-Whitney U test or Pearson’s chi-square test

Age, sex, systolic blood pressure, BMI, total cholesterol, HbA_1c_, physical activity, and serum 25(OH)D values in the 11,752 subjects successfully genotyped for rs7968585 are presented in Table 2 with no significant trends observed across the genotypes.

**Table 2:** Baseline characteristics of the 11,752 subjects in 1994 according to rs7968585 genotype. The Tromsø Study.

	T:T	T:C	C:C	Total
N	3,621	5,729	2,402	11,752
Age (years)	57.9 ± 13.6	57.8 ± 13.5	57.8 ± 13.7	57.8 ± 13.6
Sex (% females)	54.9	55.4	54.4	55.0
Current smokers (%)	34.1	34.0	33.7	34.0
Physical activity (% active)	36.8	39.2	36.8	38.0
Systolic BP (mmHg)	143 ± 23	143 ± 23	143 ± 23	143 ± 23
BMI (kg/m^2^)	25.9 ± 4.1	25.8 ± 4.1	25.8 ± 4.0	25.8 ± 4.1
Total cholesterol (mmol/L)	6.56 ± 1.32	6.53 ± 1.31	6.57 ± 1.32	6.55 ± 1.32
HbA_1c_ (%)^a^	5.44 ± 0.62	5.46 ± 0.70	5.46 ± 0.65	5.46 ± 0.66
25(OH)D (nmol/L) ^b^, smokers	72.6 ± 19.9	72.4 ± 19.9	72.9 ± 20.5	72.6 ± 20.0
25(OH)D (nmol/L) ^c^, nonsmokers	52.7 ± 17.2	52.4 ± 16.5	52.3 ± 16.2	52.5 ± 16.7

Trends across genotypes were not significant; chi-square test for linear-by-linear association, Kruskal—Wallis test or linear regression.

^a^Measured only in those attending the second visit of the Tromsø Study in 1994–1995, N = 7,182.

^b^Measured only in those attending the second visit of the Tromsø Study in 1994–1995, N = 2,334.

^c^Measured only in those attending the second visit of the Tromsø Study in 1994–1995, N = 4,826.

Among the 11,752 successfully genotyped subjects for rs7968585, 4,198 had been randomly selected for the control cohort, 1,054 subjects were registered with T2D, 2,287 subjects had confirmed MI, 3,166 had cancer, among whom there were 431 breast, 385 lung, 501 colorectal, and 406 prostate cancer cases, and 4,336 deaths. Mean follow-up time from birth was 60.8 years for T2D and MI, 61.2 years for cancer, while the mean follow-up time from examination date was 16.5 years for death. The distribution of major endpoints within the rs7968585 genotype groups is presented in Table 3, with significant trend across genotypes for T2D and MI (*P*<0.05).

**Table 3:** Distribution of endpoints in rs7968585 genotype groups. The Tromsø Study.

	Major homozygote (T:T)	Heterozygote (T:C)	Minor homozygote (C:C)	Total
	N (% within genotype)	N (% within genotype)	N (% within genotype)	N
**T2D cases** *	296 (21.1)	530 (23.7)	228 (24.7)	1,054
**T2D controls** *	1,108 (78.9)	1,707 (76.3)	694 (75.3)	3,509
**MI cases** *	680 (40.0)	1,110 (41.8)	497 (45.2)	2,287
**MI controls** *	1,020 (60.0)	1,544 (58.2)	602 (54.8)	3,166
**Cancer cases**	969 (49.8)	1,560 (50.2)	637 (50.9)	3,166
**Cancer controls**	975 (50.2)	1,547 (49.8)	615 (49.1)	3,137
**Death cases**	1,353 (66.7)	2,089 (67.0)	894 (68.8)	4,336
**Death controls**	677 (33.3)	1,027 (33.0)	405 (31.2)	2,109

*P<0.05 chi-square test for linear-by-linear association.

The HRs for all endpoints in regard to rs7968585 genotype are shown in Tables 4 and 5. Considering major homozygote (T:T) as a reference, subjects with the minor homozygote (C:C) had a significantly (*P* = 0.044 after Bonferroni correction) 25% increased risk of developing T2D in Models 1 and 2. Similarly for MI, subjects with the minor homozygotes had a 14% and 13% increased risk in Models 1 and 2, respectively, but the *P*-value was >0.05 after the Bonferroni correction for multiple testing. Furthermore, when T2D was included as a covariate, the increased risk of MI in subjects with the minor homozygote genotype was reduced to 11% (*P* = 0.080). No significant impact of the rs7968585 genotypes on the risk of total cancer (as well as for the four subtypes breast, lung, colorectal, and prostate, data not shown) and mortality was observed.

**Table 4:** Hazard ratios (HR) for the rs7968585 genotypes regarding T2D, MI, cancer and mortality analyzed with the Cox regression, adjusted for age and gender. The Tromsø Study.

	**T2D**		**MI**		**Cancer**		**Mortality**	
Endpoint (N)	1,054		2,287		3,166		4,336	
Controls (N)	3,509		3,166		3,137		2,109	
**Rs7968585 Model 1**	**HR (95% CI)**	**Unadjusted *P*-value**	**HR (95% CI)**	**Unadjusted *P*-value**	**HR (95% CI)**	**Unadjusted *P*-value**	**HR (95% CI)**	**Unadjusted *P*-value**
Major homozygote (T:T)	Reference		Reference		Reference		Reference	
Heterozygote (T:C)	1.15 (1.00–1.33)	0.054	1.04 (0.95–1.15)	0.413	1.01 (0.94–1.10)	0.732	0.99 (0.92–1.06)	0.736
Minor homozygote (C:C)	1.25 (1.05–1.49)	0.011*	1.14 (1.02–1.28)	0.023	1.07 (0.97–1.19)	0.164	1.01 (0.93–1.10)	0.861

*P<0.05 after adjusting for multiple testing, unadjusted P-value multiplied by factor of 4.

**Table 5:** Hazard ratios (HR) for the rs7968585 genotypes regarding T2D and MI analyzed with the Cox regression, adjusted for age, gender and additional risk factors. The Tromsø Study.

	**T2D**		**MI**	
Endpoint (N)	1,054		2,287	
Controls (N)	3,509		3,166	
**Rs7968585 Model 2** ^a^	**HR (95% CI)**	**Unadjusted *P*-value**	**HR (95% CI)**	**Unadjusted *P*-value**
Major homozygote (T:T)	Reference		Reference	
Heterozygote (T:C)	1.20 (1.04–1.38)	0.014	1.05 (0.95–1.15)	0.358
Minor homozygote (C:C)	1.25 (1.05–1.49)	0.011*	1.13 (1.01–1.27)	0.039
**Rs7968585 Model 3** ^b^				
Major homozygote (T:T)			Reference	
Heterozygote (T:C)			1.04 (0.94–1.14)	0.480
Minor homozygote (C:C)			1.11 (0.99–1.25)	0.080

*P<0.05 after adjusting for multiple testing, unadjusted P-value multiplied by factor of 4.

^a^Model 2 in T2D and MI groups: adjusted for age, gender, systolic BP, BMI, total cholesterol, smoking status and physical activity.

^b^Model 3 in MI: adjusted as in Model 2 and for T2D status.

There were ~8,500 subjects successfully genotyped for FokI, BsmI, TaqI, ApaI and Cdx2 groups within the control cohort and the endpoint groups. However, we did not observe significant trends across their genotypes for serum 25(OH)D status. Furthermore, none of the FokI, BsmI, TaqI, ApaI and Cdx2 genotypes showed significantly increased risk of any of the four endpoints in Cox regression analyses after Bonferroni correction (data not shown).

Serum 25(OH)D status was significantly associated with T2D, MI, and death but not cancer. When the cohort was divided according to the serum 25(OH)D 20^th^ percentile, the subjects in the low serum 25(OH)D group had an 73% increased risk of T2D (95% confidence interval (CI) 1.40–2.14, *P*<0.01), a 20% increased risk of MI (95% CI 1.02–1.41, *P*<0.05), and a 21% increased risk of death (95% CI 1.09–1.34, *P*<0.01). Same trend was observed analyzing serum 25(OH)D as a continuous variable: HR per SD decrease was 1.45 for T2D in smokers, 1.26 in nonsmokers. For MI, HR per SD decrease was 1.14 in smokers, not significant in nonsmokers. For mortality, HR per SD was 1.10 in smokers and 1.07 in nonsmokers.

The 20^th^ percentile for serum 25(OH)D was 33.9–46.6 nmol/L in nonsmokers and 50.4–64.2 nmol/L in smokers, differing according to the month. The number of subjects with serum 25(OH)D below the 20^th^ percentile (month and smoking specific) was 115, 182, 188, and 471 for the T2D, MI, cancer, and mortality case groups, respectively. However, there were no significant interactions between serum 25(OH)D status (above/below 20^th^ percentile, as well as when serum 25(OH)D was used as a continuous variable) and rs7968585 genotype regarding any of the endpoints.

In particular, for T2D, the positive association with low serum 25(OH)D (below the 20^th^ percentile compared to those above) was seen for subjects in all three rs7968585 genotypes with an increased risk of 123% for major homozygotes (95% CI 1.54–3.22, *P*<0.01) 46% for heterozygotes (95% CI 1.30–2.19, *P*<0.05), and 79% for minor homozygotes (95% CI 1.10–2.92, *P*<0.05).

## Discussion

In our large, community-based study of 8,461 subjects, we found that the minor allele (C) at rs7968585 is a significant risk factor for T2D, possibly also for MI, but not for cancer or death. To our knowledge, the association between rs7968585 and T2D is reported for the first time; meanwhile, the relation between this genotype and MI was probable, based on previous observation by Levin et al., where the risk of a composite health outcome including MI was found to be modified by the minor allele of the rs7968585 genotype in subjects with low serum 25(OH)D levels [27].

The 25% increased risk of T2D in the rs7968585 minor homozygote subjects contrasts the results from the 1958 British Birth cohort study where the *VDR* SNP rs739837 (which is in high LD with rs7968585 (r^2^ = 0.87)) was not associated with T2D [28]. In addition to the possible effect of the slight genetic difference between the two SNPs, one possible explanation could be the lack of power in the 1958 British Birth cohort study that included only 5,160 subjects. In addition, the subjects were considerably younger than in our study, and therefore, probably had fewer cases with T2D included. In support of our findings, there was a positive, although not statistically significant, association between minor homozygote and T2D.

In addition to T2D, we also observed a 14% increased risk of MI in the minor homozygote subjects. This increased risk was significant only before correction for multiple testing, but as the pathogeneses of the endpoints probably are biologically separate, the necessity for the correction might be questionable. However, when T2D was included as a confounder in the Cox regression for the risk of MI, the association between rs7968585 and MI was decreased, indicating that the genetic influence on MI was partly mediated by its effects on glucose metabolism. The association between rs7968585 and MI was also reported by Levin et al. from the Cardiovascular Health Study Discovery Cohort that included 1,514 subjects with 214 cases with MI [27]. Similar to our study, Levin et al. found a 20% increased risk for each additional minor allele, but this did not reach statistical significance. However, when viewing the two studies combined, a true association appears to be likely.

However, we could not replicate the positive association between the minor homozygote rs7968585 and cancer as well as death reported in the Cardiovascular Health Study Discovery Cohort [27]. We found only a slight nonpositive association with cancer but found no relevant association for death. Accordingly, more studies are needed to establish associations between rs7968585 and these clinical endpoints.

If such associations for rs7968585 are established, the plausible biological explanation would be through an effect on the VDR [40]. Thus, rs7968585 is located in a noncoding region close to the *VDR* gene and is in high to moderate LD with the most studied *VDR* SNPs Apal (r^2^ = 0.87), Taql (r^2^ = 0.65) and Bsml (r^2^ = 0.65) [41, 42]. The observed associations between rs7968585 and T2D as well as MI could therefore reflect covariation with these or other SNPs as well as effects on the VDR via still not proved coding of the VDR or the noncoding RNAs [43].

Thus, the nuclear receptor VDR is expressed in pancreatic cells, is involved in regulating insulin resistance, secretion, and inflammation of pancreatic islets [8, 12, 13], and may accordingly play a role in pathogenesis of T2D. The VDR is also expressed in cardiomyocytes, vessels, and other tissues involved in CAD pathogenesis, and when activated seems to protect for CAD [1, 14, 15].

Finally, the VDR seems to modify cancer-related processes by regulating cell proliferation, differentiation, and apoptosis [16, 17]. In line with this, the commonly studied *VDR* SNP FokI (in low LD with rs7968585) appears to be associated with T2D, at least in Asian populations [13, 18] and cancer [44, 45]; and the SNPs ApaI, BsmI, as well as TaqI appear to be associated with CAD [22–24] as well as with cancer [44, 45] and cancer-related mortality [21]; and some Cdx2 (in low LD with rs7968585) haplotypes appear to be associated with increased risk of cancer [46]. However, in our study, none of the FokI, BsmI, TaqI, ApaI, Cdx2 SNPs showed any significant associations with the risk for these outcomes, which might be due to inter-population genetic variances.

Another major objective of our study was to evaluate possible interactions between rs7968585 and the serum 25(OH)D levels as described by Levin et al. regarding their composite endpoint [27]. This interaction is plausible given that the rs7968585 minor allele makes the VDR less active. If so, for subjects with minor alleles, the presumed positive effects by the VDR would need higher serum 25(OH)D levels to be activated [40]. However, in spite of the expected significant associations between serum 25(OH)D and subsequent T2D, MI and death [1, 6], no interactions between rs7968585 and serum 25(OH)D levels regarding the clinical endpoints were found, which might be due to generally sufficient serum 25(OH)D levels in our population (48.5–52.7 nmol/L in nonsmokers) in contrast to the study of Levin et al. [27]. Thus, in our study the negative effects of the minor allele were seen regardless of serum 25(OH)D levels, and a similar conclusion was reached by Vimaleswaran et al. regarding serum 25(OH)D and T2D in their British Birth cohort study [28]. One probable explanation could be that rs7968585 acts through modulating the response to vitamin D, independently from vitamin D status as suggested by Barry et al. [47]. However, given the attractiveness of such an interaction between serum 25(OH)D and the *VDR*, this should be tested not only for rs7968585 but also in all studies in which both genetic as well as serum 25(OH)D data are available.

Our study has several limitations. Firstly, after the Bonferroni correction, the association between rs7968585 and the endpoints was significant only for T2D. This may reflect that for this type of study even a cohort of more than 8,000 subjects may be too small. Secondly, not all subjects had serum 25(OH)D measurements, which substantially reduced the power for the interaction part of the study. Thirdly, we relied on only one serum 25(OH)D measurement, and even though there is a high degree of tracking for serum 25(OH)D [48], several consecutive measurements would have been a great advantage. Finally, we do not know the consequences of the rs7968585 SNP for the function of the VDR, and it is therefore difficult to fit this SNP into a biological context.

Our study also has several strengths. The endpoint registers were of high quality and community-based in a large north Norwegian population with long-term follow-up. We also had the opportunity to take advantage of a previous publications regarding rs7968585 and therefore focused on this SNP only, which increased the statistical power.

In conclusion, we found a significant association between the *VDR* SNP rs7968585 and risk of T2D and possibly also MI in a general population, whereas interactions between rs7968585 and serum 25(OH)D levels regarding clinical endpoints were not found. Confirmatory studies are needed.
