# Correction: Genetic Variations in the Vitamin D Receptor Predict Type 2 Diabetes and Myocardial Infarction in a Community-Based Population: The Tromsø Study

**DOI:** 10.1371/journal.pone.0163573

**Published:** 2016-09-19

**Authors:** Ieva Martinaityte, Rolf Jorde, Henrik Schirmer, Ellisiv Bøgeberg Mathiesen, Inger Njølstad, Maja-Lisa Løchen, Tom Wilsgaard, Ragnar Martin Joakimsen, Elena Kamycheva

The first author’s name is incorrect. The correct name is Ieva Martinaityte. The correct citation is: Martinaityte I, Jorde R, Schirmer H, Mathiesen EB, Njølstad I, Løchen M-L, et al. (2015) Genetic Variations in the Vitamin D Receptor Predict Type 2 Diabetes and Myocardial Infarction in a Community-Based Population: The Tromsø Study. PLoS ONE 10(12): e0145359. doi:10.1371/journal.pone.0145359
